# Aldose Reductase Inhibition by *Orthosiphon stamineus* Extracts and Constituents Suggests Antioxidant Potential

**DOI:** 10.3390/molecules30234637

**Published:** 2025-12-02

**Authors:** Yousaf Dawood, Atheer Zgair, Mun Fei Yam, Nur Hidayah Kaz Abdul Aziz

**Affiliations:** 1 School of Pharmaceutical Sciences, Universiti Sains Malaysia, Minden 11700, Malaysia; ph.yousifdawood@uoanbar.edu.iq (Y.D.); yammunfei@usm.my (M.F.Y.); 2 College of Pharmacy, University of Anbar, Ramadi 31001, Iraq

**Keywords:** *Orthosiphon stamineus*, aldose reductase, AKR1B1, oxidative stress, antidepressant, eupatorin, sinensetin, rosmarinic acid

## Abstract

Background/Objectives: Aldose reductase (AR) plays a crucial role in the accumulation of oxidative factors that lead to oxidative stress-related neuroinflammation. This study aims to find a novel agent from natural sources that can inhibit AR. Methods: Different extracts of *Orthosiphon stamineus* Benth (OS) leaves and its active constituents, eupatorin (EUP), rosmarinic acid (RA), sinensetin (SEN) and 3-hydroxy-5,6,7,4-tetramethoxyflavone (TMF), were used to identify the potential inhibition effect of AR. A new high-performance liquid chromatography (HPLC) method was developed to determine these phytochemicals using the Shimadzu LC-20AD HPLC system. In addition, the in vitro inhibition effect of OS ethanol extracts (95% and 50%) and OS components EUP, RA, and SEN was investigated in recombinant AR (AKR1B1). Results: In this study, the developed HPLC method was precise and accurate, and demonstrated clear separation of the four compounds—EUP, RA, SEN, and TMF—in the ethanolic extract. The contents of the four selected compounds—EUP, RA, SEN, and TMF—in 95% ethanolic extract were 2.35, 11.91, 0.94, and 0.18%, respectively. RA showed the highest concentration among the selected compounds, indicating that RA is the major constituent of this plant. The in vitro assay showed significant inhibition of the AR enzyme by RA and OS ethanol extracts 95% and 50% (IC_50_: 41.42 µM; 63.42 µg/mL and 93.22 µg/mL, respectively). Conclusions: The ethanolic extract of OS and RA could be a promising therapeutics option for the treatment of oxidative stress-related neuroinflammation disorders by inhibiting AR.

## 1. Introduction

*Orthosiphon stamineus* Benth (OS) is a traditional Asian herbal remedy, known for its wide range of therapeutic applications, such as the treatment of inflammation, bacterial infections, diabetes, urinary tract infections, influenza, rheumatism, jaundice, and cancer-related conditions involving angiogenesis [1]. Numerous studies have demonstrated the ability of the OS plant to counteract inflammation and oxidative stress, which are considered important factors implicated in central nervous system diseases [2,3]. Chung et al. showed that proteins derived from OS leaves exhibited neuroprotective properties against H_2_O_2_-induced stress and have anticonvulsant effects in both normal and hypoxic–ischemic situations [4]. In addition, a recent report demonstrated that the ethanolic leaf extract of OS exhibited promising efficacy in treating Alzheimer’s disease-like symptoms by effectively reversing memory impairment [5]. However, studies investigating the neuroprotective effect of OS are still limited. The wide spectrum of therapeutic properties of OS is largely attributable to the complexity and diversity of its phytochemical constituents. Several components of this herb have been isolated, such as phenolics, flavonoids, terpenoids, organic acids, and essential oils [6]. However, most of the therapeutic action of this plant is attributed to certain active constituents, including eupatorin (EUP), rosmarinic acid (RA), sinensetin (SEN), and 3-hydroxy-5,6,7,4-tetramethoxyflavone (TMF) [7,8]. In an unpublished study, these four selected compounds were analyzed for potential targets using the SwissTargetPrediction server (www.swisstargetprediction.ch, accessed on 2 May 2024). Interestingly, the aldose reductase (AR) enzyme was predicted to be the most likely target for all the selected compounds derived from OS.

The AR enzyme, which belongs to the keto-reductase family (AKR1B1, in humans), converts glucose to sorbitol. The accumulation of sorbitol in the tissue results in osmotic stress [9]. In addition, it has been documented that the AR enzyme can reduce glutathione-conjugated lipid aldehyde (GS-HNE) to glutathione-conjugated dihydroxynonanal (GS-DHN). The latter has been demonstrated to play a pivotal role in the activation of protein kinase C (PKC), subsequently initiating the transcription of several inflammatory mediators such as cytokines, TNF-α, and iNO [10]. The increased generation of reactive oxygen species (ROS) and subsequent activation of pro-inflammatory pathways have an impact on the pathophysiology of several oxidative stress-related diseases [11]. Numerous studies have found that suppressing this enzyme will disrupt ROS signaling and decrease the progression of the inflammatory response [12,13]. Inhibition of AR with sorbinil has been shown to provide a neuroprotection effect against Alzheimer’s disease by reducing the release of inflammatory mediators, increasing phagocytosis, and modulating microglial migration [14].

In addition, there are many natural compounds, in particular flavonoids such as luteolin and quercetin or plant extracts such as Indian gooseberry, spinach, basil, and cinnamon, were reported to inhibit AR. These natural AR inhibitors can inhibit oxidative stress, reduce inflammation variables, and aid in the prevention of diabetic complications and even other disorders related to oxidative stress [15]. Goodarzi et al. (2006) found that quercetin strongly inhibits the aldose reductase enzyme with an IC_50_ value of 5 µM [16]. Interestingly, quercetin has been found in other recent works to exert its antidepressant effect by inhibiting neuronal inflammation induced by glial cell activation and oxidative stress [17]. Notably, AR contributes to microglial activation, promoting the release of pro-inflammatory cytokines and phagocytic activity. These processes exacerbate neuroinflammation and synaptic injury, potentially leading to major depressive disorders [11]. Therefore, we hypothesized that the inhibition of AR could serve as a pharmacological strategy to attenuate stress-induced neuroinflammation and depression. The present findings suggest that OS extract and its active constituents may inhibit AR-mediated oxidative processes. Accordingly, this study aimed to develop a validated HPLC method for the determination of OS major constituents and to evaluate the inhibitory effects of OS extracts and their principal constituents on AR enzyme activity.

## 2. Results

### 2.1. HPLC Analysis

#### 2.1.1. Selectivity

**Figure 1:**
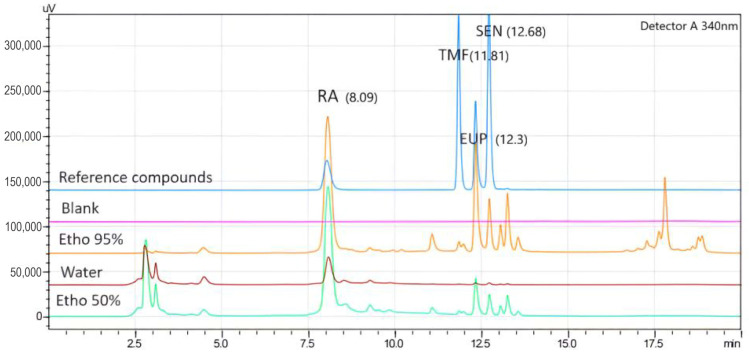
Chromatogram of *Orthosiphon stamineus* extracts and reference compounds; EUP, eupatorin; RA, rosmarinic acid; SEN, sinensetin; TMF, 3-hydroxy-5,6,7,4-tetramethoxyflavone; Etho 95% and Etho 50%, OS ethanol extracts (95% and 50%).

In the present study, markers were detected using the gradient method in less than 20 min. The peaks of EUP, RA, SEN, and TMF were verified by comparing their retention time with the reference standards. EUP, RA, SEN, and TMF were eluted at 12.30, 8.09, 12.68, and 11.81 min, respectively (Figure 1). The response of the reference compounds was five times higher compared to the blank at the lower limit of quantification (LLOQ), which was 15.62 µg/mL for RA and 1.95 µg/mL for TMF, EUP, and SEN. The relative standard deviation (RSD) and the relative error (RE) were within acceptable limits according to the FDA guidelines [18].

#### 2.1.2. Linearity

Linearity was observed at concentrations of 1.95 to 250 µg/mL with r^2^ values equal to or greater than 0.99 in all calibration curves. Table 1 presents reference compounds’ intra- and inter-day precision and accuracy data. All RSD and RE values were less than or equal to 15%.

#### 2.1.3. Quantification of RA, TMF, EUP, and SEN from Different OS Extracts

The developed HPLC method was used to determine the selected compounds—EUP, RA, SEN, and TMF—in three different extracts of OS. The chromatograms for both 95% ethanol and 50% ethanol extracts showed effective separation of all compounds. However, the water extract showed obvious separation for RA only as shown in Figure 1. Results are presented as average *w*/*w*% ± SD (Table 2). The 95% ethanolic extract contained the highest amount of marker compounds compared to the 50% ethanolic extract and water extract. RA exhibited the highest concentration among the selected markers in all extracts, particularly in the 95% ethanolic extract.

### 2.2. In Vitro AR Inhibition Assay

Aldose reductase is recognized as the rate-limiting enzyme in the polyol pathway and a key contributor to the generation of oxidative stress. Consequently, its inhibition has emerged as an important therapeutic strategy for managing oxidative stress-related disorders. In the present study, ethanolic extracts of OS and its main active constituents (EUP, RA, and SEN) demonstrated inhibitory effects on the AKR1B1 isoform of AR. The percentage of inhibition of all tested compounds showed a concentration-dependent decrease. Among all compounds tested, RA exhibited the strongest inhibitory activity. Notably, its lowest tested concentration (17.3 µM) produced a significant inhibition effect (23.2%, *p* < 0.01). By contrast, EUP and SEN required higher concentrations at approximately 36 and 67 µM, respectively, to achieve significant inhibition effects (10.77% and 8.98%, respectively). Significant inhibitory effects were also observed in 95% and 50% ethanolic OS extracts (33.87% and 35.75%, respectively) at concentrations of 31.25 and 62.5 µg/mL, respectively.

**Figure 2:**
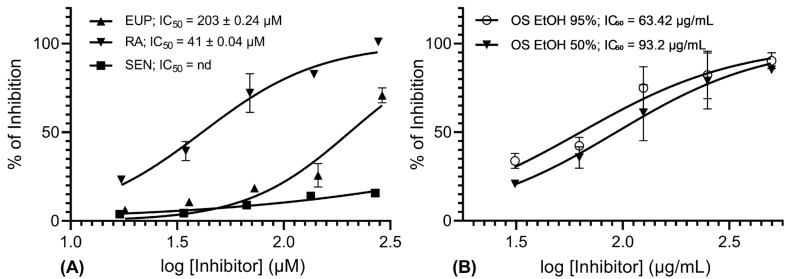
AKR1B1 inhibition dose–response curve for (**A**) phytochemicals (EUP, eupatorine; RA, rosmarinic acid; and SEN, sinensetin) and (**B**) *Orthosiphon stamineus* (OS) ethanolic extracts (EtOH 95% and 50%). The data are shown as mean ± SD of % of inhibition (*n* = 3). IC_50_ values were calculated using non-linear regression (4-parameter logistic curve).

The IC_50_ value of EUP was 203 ± 0.24 µM, while RA displayed a substantially lower IC_50_ of 41.4 ± 0.04 µM, indicating greater potency. SEN exhibited only weak and poorly quantifiable inhibition, excluding the determination of its IC_50_ (Figure 2A). The OS ethanolic extracts yielded distinct IC_50_ values, with 95% ethanol extract showing greater potency (IC_50_ = 63.42 µg/mL) than the 50% ethanol extract (IC_50_ = 93.2 µg/mL), as presented in Figure 2B.

## 3. Discussion

This study detailed how OS ethanol extracts and their main active constituents, RA, EUP, and SEN, suppress AKR1B1, an enzyme linked to oxidative stress that plays a crucial role in the progression of inflammatory disorders.

In this study, a validated HPLC method was successfully developed. The gradient elution method, which employed different types of mobile phases at column temperature of 40 °C, allowed the separation of poorly water-soluble compounds such as EUP, SEN, and TMF from the water-soluble RA. This simultaneous separation improves the reliability of standardization and enables the accurate quantification of each compound in various extraction solvents. In contrast, previous methods were not always able to efficiently separate all these compounds in a single run or required harsh mobile phase conditions that could overwhelm the lifetime of the column [19]. Furthermore, this method separated all the tested compounds in a short run time (less than 20 min), representing a major breakthrough compared to previous methods [20]. This short run time saves both cost and time, making it very appropriates for the routine quality control of herbal products.

The 95% ethanol extract was found to have the highest levels of these bioactive compounds, particularly RA, indicating that OS leaves contain substantial amount of this substance. However, the content of TMF was the lowest compared to other constituents. These findings were evidenced by other investigators who identified RA as a major compound in OS and highlighted its antioxidant and anti-inflammatory activities [21,22]. To note, the recovery of RA in the current study was higher than that reported by Yehya and colleagues, who used Nuvastatic™ (C5OSEW5050ESA), a standardized RA-rich extract [21]. The discrepancy in the results of recoveries might stem from different factors such as chromatographic conditions, extraction process, and the origin of the plant [22].

Water extraction of OS showed minimal presence of hydrophobic flavonoids, EUP, SEN, and TMF. This is likely due to their poor water solubility, confirming that aqueous extraction is optimal for water-soluble constituents such as RA. Other investigators also reported similar findings [20,22]. Consequently, the water extract and TMF were excluded from the in vitro analysis of the current study.

The AR inhibition assay revealed that OS 95% ethanol extract significantly inhibited AR activity, with an IC_50_ of 63.42 µg/mL. This extract exhibited higher inhibition than the 50% ethanol extract (IC_50_ = 93.20 µg/mL), reflecting a higher concentration of active constituents. Of the compounds evaluated, RA exhibited the most significant inhibitory effect against AR, with an IC_50_ of 41.4 µM. This supports the hypothesis that RA is a major contributor to the inhibitory effect of OS.

Although EUP also suppressed AR (IC_50_ = 203 µM), its inhibition was much less effective compared to RA. In contrast, SEN showed only very limited inhibition in the concentrations tested and is hence not suitable for IC_50_ determination. Such divergences may arise from differences in molecular structures, binding affinities, and physicochemical properties.

It is also worth considering the potential synergistic interactions between RA and other flavonoids such as EUP, which may enhance the overall inhibitory effect of the extracts. However, this requires further mechanistic investigation using combination index analysis or molecular docking.

The findings in the current study are consistent with earlier pharmacological reports on OS and RA, which demonstrated anti-inflammatory and neuroprotective effects [4,5]. Thus, although the in vitro IC_50_ of the RA and OS extract is relatively high and may not be achieved physiologically, they provide relevant preliminary information on the effect of OS, indicating that it is an effective AR inhibitor.

## 4. Materials and Methods

### 4.1. Chemicals and Reagents

The dried OS leaves were purchased from a local supplier (Herbagus, Penang, Malaysia). EUP (CAS: 855-96-9), RA (CAS: 20283-92-5), SEN (CAS: 2306-27-6), TMF (CAS 65548-55-2), and Epalrestat were purchased from Sigma-Aldrich (Selangor, Malaysia). The recombinant human AKR1B1 protein (His Tag) was bought from Elabscience company (Houston, TX, USA). DL-glyceraldehyde was obtained from Nacalia Tesque (Kyoto, Japan). Furthermore, beta-nicotinamide adenine dinucleotide phosphate tetrasodium (NADPH) was acquired from Tokyo Chemical Inc. (Tokyo, Japan).

### 4.2. Standard and Sample Preparation

The dried *OS* leaves were crushed using a 60 mesh-size 10-horsepower Pulverizer machine. The herbal powder was packed in sealed opaque bags in 100 g aliquots, and the maceration method was used for extraction. The ground leaves (100 g) were soaked in 1 L of water, 95% ethanol, or 50% ethanol at 60 °C for 8 h. The soaking process was repeated three times. The supernatants were then collected and filtered using Whatman filter paper (No. 1). The filtrate was concentrated and dried using a rotary evaporator, which has previously been demonstrated as an efficient drying method for OS, while also preserving its phytochemical content [23,24]. Finally, the crude extracts obtained, 2 mg each, were dissolved in 1 mL of methanol and filtered using a 0.45 µm syringe filter.

In addition, one milligram of each standard compound, EUP, RA, SEN, and TMF, was dissolved in 1 mL of methanol. The stock solutions were then serially diluted with methanol. These solutions were filtered using a 0.45 µm syringe filter and sonicated for 15 min.

### 4.3. Identification and Quantification of EUP, RA, SEN, and TMF in OS Extracts

#### Chromatographic Conditions

The Shimadzu LC-20AD HPLC system (Shimadzu Corporation, Kyoto, Japan) was used for analysis. The separation was carried out on a Thermo Scientific^®^ C18 column (250 × 4.6 mm, 5 µm particle size), and the column temperature was set at 40 °C. The mobile phase was composed of (A) 20 mM phosphate buffer (at pH 3), (B) acetonitrile, and (C) methanol with gradient elution as described in Table 3. The flow rate was tuned to 1 mL/min, and the UV detector wavelength was 340 nm. The run time was 20 min, and the injection volume was 5 µL.

### 4.4. Method Validation

The current method was validated according to the FDA guidelines for bioanalytical method validation focusing on selectivity, linearity, precision, and accuracy [18].

#### 4.4.1. Selectivity

Selectivity was determined by comparing blank chromograms from six different batches with the mixture of standard compounds spiked at the lower limit of quantification (LLOQ; *n* = 6).

#### 4.4.2. Sensitivity and Linearity

The LLOQ of this method was determined as the lowest concentration of injected standard that met the criteria of relative error (RE) within ±20% and relative standard deviation (RSD) no greater than 20% for intra-day analyses [18]. To evaluate the linearity, graphical plots of peak areas versus the concentration range 1.95–250 µg/mL of the standards were created. Linearity was presented by correlation coefficient analysis (r^2^).

#### 4.4.3. Intra- and Inter-Day Precision and Accuracy

RSD and RE values were used to determine intra-day precision and accuracy, respectively. To assess intra-day precision and accuracy, four replicates of each quality control (QC) sample (Table 4) were injected on one day. Inter-day precision and accuracy were measured by injecting QC samples on five different days (Table 4). The method was considered precise and accurate when RSD < 15% and RE < ±15% for both intra- and inter-day runs of QC samples.

#### 4.4.4. Quantification of EUP, RA, SEN, and TMF from Different OS Extracts

Different extracts (ethanol 95%, ethanol 50%, and water) of *OS* were injected at 2 mg/mL, and the peak areas corresponding to EUP, RA, SEN, and TMF were recorded. The concentration of these compounds in the extracts was then measured by applying the linear regression equations of the standard calibration curves (*n* = 5). These four marker chemicals were represented as a *w*/*w* percentage (% *w*/*w*) of the dried extract.

### 4.5. In Vitro AR Inhibition Assay

In this study, the ethanolic extracts of OS (95% and 50%), EUP, RA, and SEN were dissolved in 10% DMSO to prepare the stock solutions. The enzyme activity was measured using a method modified by Miláčková and coworkers [25]. Briefly, 20 µL of each concentration of the samples were added into respective wells, followed by 110 µL of sodium phosphate buffer, 20 µL of D, L-glyceraldehyde, and 40 µL of NADPH added into each well, and incubated for 5 min. The final concentrations of the samples consist of five serial concentrations ranging from 31.25 to 500 µg/mL of 50% and 95% ethanolic extracts; 18 to 290 µM EUP; 17 to 278 µM RA; and 17 to 270 µM SEN. Then, AKR1B1 (10 µL) was added to bring the final volume to 200 µL and initiate the enzymatic reaction. In addition, final concentrations of 0.5 µM Epalrestat and 1% DMSO were used as positive and negative control wells, respectively. All standards and samples were tested in triplicates. The absorbance was measured using a multimode microplate reader (TECAN^®^, Männedorf, Switzerland) at 340 nm every minute for up to 4 min duration to monitor the consumption of NADPH. The enzyme’s activity was measured as a percentage of the control (100%), as described in Equation (1), and IC_50_ values (the concentration of inhibitor required to reduce enzyme activity by 50%).

(1) $$\%\;\mathrm{inhibition}=\left(\frac{∆OD\;of\;Control-∆OD\;of\;sample}{∆OD\;of\;control}\right)\times 100$$

where ∆OD is the difference in optical density.

### 4.6. Statistical Analysis

Data was analyzed using GraphPad Prism software (version 8.0.0 for Windows, GraphPad Software, San Diego, CA, USA). Disparities among treatment groups were assessed using ordinary one-way ANOVA, followed by Dunnett’s multiple comparisons test to identify significant differences between inhibitors. IC_50_ values were determined using non-linear regression (4-parameter logistic curve). Results are presented as mean ± SD, where *p* < 0.05 denotes statistical significance.

## 5. Conclusions

This study provides new evidence supporting the therapeutic potential of OS in targeting oxidative stress-related disorders through the inhibition of the AR enzyme. A validated HPLC method was successfully developed to simultaneously determine RA, EUP, SEN, and TMF in OS extracts. RA was identified as the most abundant component.

In addition, the 95% ethanolic extract of OS, which contained the highest concentration of active constituents, exhibited significant inhibitory activity against aldose reductase (AR/AKR1B1). RA demonstrated the most potent inhibition, suggesting that RA is likely the major contributor to the AR-inhibitory effect of OS. To our knowledge, this is the first study to directly evaluate AR-inhibitory activity of OS and its isolated constituents in vitro, thus contributing valuable new insight into the pharmacological potential of this medicinal plant.

These findings indicate that OS, particularly its ethanolic extract and RA component, may serve as promising natural agents for developing novel therapies targeting AR-mediated oxidative pathways.

Addressing the possible synergistic or additive effect of RA and EUP combination on AR inhibition in future investigations is essential. In addition, further in vivo research is needed to clarify their potential clinical applications and underlying mechanisms in relation to depression.

## Figures and Tables

**Table 1:** Intra- and inter-day precision and accuracy data for the determination of EUP, RA, SEN, and TMF in different extracts of *Orthosiphon stamineus* (OS).

Marker	Quality Control	Level (µg/mL)	Intra-Day (*n* = 5)		Inter-Day (*n =* 5)	
			RSD%	RE%	RSD%	RE%
Rosmarinic acid (RA)	LLOQ	15.62	1.79	−16.86	2.07	−18.35
	LQC	31.25	0.09	−8.35	0.06	−9.47
	MQC	125	0.20	4.57	0.05	4.25
	HQC	250	0.11	0.12	1.38	−1.15
3′-hydroxy-5,6,7,4′-tetramethoxyflavone (TMF)	LLOQ	1.95	2.09	18.21	2.80	20.26
	LQC	15.62	0.77	−0.10	1.17	−0.22
	MQC	31.25	0.18	−2.40	1.75	−1.60
	HQC	125	0.63	1.55	0.91	1.27
Eupatorin (EUP)	LLOQ	1.95	3.75	−2.14	3.04	1.35
	LQC	15.62	0.61	2.64	1.11	2.85
	MQC	31.25	0.77	1.04	1.62	1.03
	HQC	125	1.05	1.82	0.78	1.82
Sinensetin (SEN)	LLOQ	1.95	3.00	−1.21	2.43	9.29
	LQC	15.62	0.69	14.89	1.32	15.88
	MQC	31.25	0.20	8.59	1.42	6.95
	HQC	125	0.59	−7.04	1.01	−7.60

RSD, relative standard deviation; RE, relative error; LLOQ, lower limit of quantification; LQC, MQC, and HQC, lower, medium, and high-quality controls, respectively.

**Table 2:** Concentration of rosmarinic acid (RA), 3′-hydroxy-5,6,7,4′-tetramethoxyflavone (TMF), eupatorin (EUP), and sinensetin (SEN) in different extracts of *Orthosiphon stamineus* Benth (OS).

	Content % (*w*/*w*) ± SD (*n* = 5)			
Extract	RA	TMF	EUP	SEN
95% ethanolic	11.91 ± 0.75	0.18 ± 0.019	2.35 ± 0.040	0.94 ± 0.050
50% ethanolic	10.60 ± 0.11	0.05 ± 0.021	0.84 ± 0.014	0.32 ± 0.009
Water	2.38 ± 0.07	nd	0.03 ± 0.003	nd

EUP, eupatorine; RA, rosmarinic acid; SEN, sinensetin; TMF, 3-hydroxy-5,6,7,4-tetramethoxyflavone; nd, not detected.

**Table 3:** Gradient elution system for the separation of *Orthosiphon stamineus* Benth (OS) constituents.

Time	Solvent Ratio (%)		
	(A) Phosphate Buffer	(B) Acetonitrile	(C) Methanol
0	80	10	10
5	50	30	20
10	20	25	55
12	0	30	70
15	45	15	40
18	80	10	10
20	80	10	10

**Table 4:** Quality control concentrations used for the validation of the HPLC method.

Markers	Level	Concentration of Standard (µg/mL)
Rosmarinic acid	LLOQ	15.62
	LQC	31.25
	MQC	125
	HQC	250
3′-hydroxy-5,6,7,4′-tetramethoxyflavone	LLOQ	1.95
	LQC	15.62
	MQC	31.25
	HQC	125
Eupatorin	LLOQ	1.95
	LQC	15.62
	MQC	31.25
	HQC	125
Sinensetin	LLOQ	1.95
	LQC	15.62
	MQC	31.25
	HQC	125

LLOQ, lower limit of quantification; LQC, MQC, and HQC; lower, medium, and high-quality controls, respectively.

## Data Availability

Data contained within the article are available from the authors.
